# Preoperative Neutrophil-Lymphocyte and Lymphocyte-Monocyte Ratios Reflect Immune Cell Population Rearrangement in Resectable Pancreatic Cancer

**DOI:** 10.1245/s10434-016-5634-0

**Published:** 2016-10-21

**Authors:** Marek Sierzega, Marzena Lenart, Magdalena Rutkowska, Marta Surman, Bozenna Mytar, Andrzej Matyja, Maciej Siedlar, Jan Kulig

**Affiliations:** 1 First Department of General and GI Surgery, Jagiellonian University Medical College, Krakow, Poland; 2 Department of Clinical Immunology, Institute of Pediatrics, Jagiellonian University Medical College, Krakow, Poland; 3 Laboratory of Clinical Immunology, University Children’s Hospital, Krakow, Poland

## Abstract

**Background:**

Neutrophil-lymphocyte ratio (NLR), platelet-lymphocyte ratio (PLR), and lymphocyte-monocyte ratio (LMR) may serve as a simple index of the immune function. The aim of this study was to investigate the prognostic significance of NLR, PLR, and LMR in patients with resectable pancreatic ductal adenocarcinoma (PDAC) and to verify whether such biomarkers are associated with changes in populations of lymphoid cells.

**Methods:**

The prognostic implications of blood count parameters were evaluated in a *retrospective cohort* of 442 subjects undergoing pancreatic resections for PDAC. Subpopulations of lymphocytes and monocytes in peripheral blood were identified by FACS in a *prospective cohort* of 54 patients.

**Results:**

In the univariate analysis, NLR < 5 and LMR ≥ 3 were associated with significantly longer median survival of 25.7 vs 12.6 months and 29.2 vs 13.1 months, respectively. PLR did not influence survival. The Cox proportional hazards model showed that high NLR (HR 1.66, 95 % CI 1.12 to 2.46, *P* = 0.012) and low LMR (HR 1.65, 95 % CI 1.06 to 2.58, *P* = 0.026) were independent predictors of poor prognosis. NLR ≥ 5 and LMR < 3 correlated with an approximately twofold decrease in counts of helper and cytotoxic T cells, B cells, and NK cells. High NLR was also accompanied with increased neutrophil counts, while low LMR showed increased numbers of monocytes, mostly classical.

**Conclusions:**

NLR and LMR may carry important prognostic information for patients with resected PDAC. The unfavorable prognosis likely correlates with reduced numbers of immune cells effective against the tumor and increased populations of cells involved in immune suppression.

The functional status of the immune system significantly affects the prognosis of many human malignancies, including gastrointestinal cancers.^1^ The tumor-related response of a host defense system is associated with rearrangements of various hematological components, such as white blood cells, specifically the neutrophils, lymphocytes and monocytes. Recently, several investigators suggested that variables derived from routine blood count parameters, i.e. platelet-lymphocyte ratio (PLR) and neutrophil-lymphocyte ratio (NLR), could serve as a simple index of the immune function and may be of prognostic significance in patients with solid tumors.^2^,^3^ This observation is of considerable clinical importance as many laboratory tests used previously to examine the inflammatory machinery are expensive or labor-intensive, and thus not suitable for routine clinical practice.

High values of two parameters incorporating lymphocyte count, i.e. NLR and PLR, have been previously reported as possibly associated with impaired survival of patients with resectable pancreatic cancer.^4^,^5^ Although the proposed biomarkers might prove useful in stratifying patients with good and poor prognosis, they have not yet been appropriately validated for clinical decision making as almost all of these studies recruited relatively small populations.^6^,^7^ Moreover, it is not known what are the mechanisms implicated in the correlation between blood counts and patients’ survival.

The aim of this study was to investigate the prognostic significance of routine lymphocyte-based blood count parameters, such as neutrophil-lymphocyte ratio, platelet-lymphocyte ratio, and lymphocyte-monocyte ratio in patients with resectable pancreatic cancer. Moreover, we sought to verify whether prognostically important values of such biomarkers are associated with changes in populations of lymphoid cells.

## Materials and Methods

### Patients

The study was carried out in 2 separate cohorts of treatment naïve patients with pancreatic ductal adenocarcinoma (PDAC). The prognostic implications of blood count parameters were evaluated in a *retrospective cohort* including subjects undergoing pancreatic resections between 1990 and 2012 for PDAC at our academic tertiary surgical center. A *prospective cohort* for experiments examining populations of immune cells in peripheral blood consisted of a group of 54 consecutive PDAC patients recruited between 2012 and 2014. Patients with prior neoadjuvant treatment as well as active infection, hematological disorders, acute and chronic inflammatory or autoimmune diseases, or prior steroid therapy were excluded. All data were prospectively collected and recorded in a dedicated database. Variables potentially affecting survival were retrieved from the database and analyzed retrospectively, including demographic data, pathologic features of the tumor, and therapeutic interventions. The extent of surgery was classified as defined by the recent guidelines.^8^,^9^ Tumors were evaluated according to the current American Joint Committee on Cancer (AJCC) classification criteria.^10^ R0 resections were assumed if all tumor-free margins were at least 1 mm wide.^11^ The study was approved by the Bioethics Committee of Jagiellonian University.

### Blood Count Parameters

All samples of peripheral blood were collected within 10 days prior to surgery (prospective and retrospective cohorts). The patients were required to have preoperative blood counts along with differential white blood cell count, including neutrophils, lymphocytes, monocytes, and platelets. NLR and PLR were calculated as the absolute count of neutrophils and platelets, respectively, divided by the absolute lymphocyte count. Similarly, LMR was defined as the ratio of the lymphocyte count to the absolute count of monocytes.

### Identification of Lymphatic Cells in Peripheral Blood

Peripheral whole blood samples were collected in sterile BD Vacutainer^®^ EDTA (ethylenediamine-tetraacetic acid) blood collection tubes (BD Biosciences). The samples were incubated in BD Trucount™ tubes (BD Biosciences) with the following monoclonal antibodies to identify lymphocyte sub-populations (BD Biosciences): anti-CD45-PerCP (clone 2D1), anti-CD3-FITC (clone UCHT-1), anti-CD4-APC (clone RPA-T4), anti-CD8-PE (clone RPA-T8), anti-CD16-PE (clone 3G8), anti-CD19-APC (clone HIB19), anti-CD56-PE (clone NCAM16.2). The following antibodies were used for monocyte sub-populations (BD Biosciences): anti-CD45-APC (clone 2D1), anti-HLA-DR-PerCP (clone L243), anti-CD14-FITC (clone M5E2), and anti-CD16-PE (clone 3G8). The samples were incubated at 4 °C for 30 min and then were treated with FACS Lysing Solution (BD Biosciences) until the erythrocytes were lysed and the cells were immediately processed in the FACSCanto flow cytometer (Becton–Dickinson Immunocytometry Systems, Palo Alto, CA) along with 10,000 beads per tube. The results were analyzed with FACSDiva Software (BD Biosciences) and the absolute numbers of lymphocytes and monocytes subsets were calculated on the basis of bead counts. Monocyte populations were classified as classical (CD14++ CD16−), intermediate (CD14++ CD16+), and non-classical (CD14+ CD16++).^12^

### Statistical Analysis

All continuous variables are reported with their median and interquartile range (IQR) while categorical data are reported as proportions. Statistical significances of the differences in categorical and continuous variables were analyzed by χ^2^ and Mann–Whitney *U* tests where appropriate. Survival data was analyzed according to the Kaplan–Meier method and included postoperative mortality. The prognostic value of each blood count parameter was tested as raw, continuous data by the Cox regression analysis and as categorical variables with the optimum cut-off values identified with the time-dependent ROC curves analysis.^13^ Multivariate analysis was performed using a Cox proportional hazards model with a backward stepwise selection procedure. The probability for entering the model was 0.05 and for removal from the model 0.100. All tests were two-sided and *P* < 0.050 was considered statistically significant. Statistical analysis was performed using the IBM^®^ SPSS^®^ Statistics 20 software package (IBM Corporation, NY).

## Results

### Patient Characteristics

A total of 536 patients who underwent pancreatic resections for PDAC were identified in the database between 1990 and 2012. Detailed blood count parameters were available in 442 patients, and they constituted the final population for the *retrospective cohort* (Table 1). There were 260 males and 182 females with a median age of 60 years (range 21–83). The *prospective cohort* recruited 54 patients with similar clinicopathologic characteristics. There were no significant differences between these two cohorts except for higher proportions of patients subject to pylorus-preserving pancreaticoduodenectomy and postoperative chemotherapy in the prospective group. No neoadjuvant therapy was used in either population and all the patients were free of recent or ongoing infection based on clinical signs and symptoms along with laboratory parameters.

**Table 1:** Clinicopathologic characteristics of the study population

Parameter	Cohort	
	Retrospective (*n* = 442)	Prospective (*n* = 54)
Age, years (median, IQR)	60 (55–66)	59 (50–68)
Female/male (*n*, %)	182 (41)/260 (59)	25 (46)/29 (54)
Body mass index (median, IQR)	23.3 (21.3–26.1)	22.6 (20.4–26.9)
WBC, 10^9^/L (median, IQR)	6.915 (5.745–8.560)	7.725 (5.930–8.430)
Neutrophils, 10^9^/L (median, IQR)	4.25 (3.36–5.67)	5.20 (3.30–6.40)
Lymphocytes, 10^9^/L (median, IQR)	1.75 (1.28–2.22)	1.64 (1.20–2.07)
Monocytes, 10^9^/L (median, IQR)	0.590 (0.480–0.850)	0.700 (0.500–0.900)
Platelets, 10^9^/L (median, IQR)	249 (200–326)	248 (198–319)
Neutrophil-lymphocyte ratio (median, IQR)	3.0 (2.0–4.1)	2.9 (2.2–3.7)
Lymphocyte-monocyte ratio (median, IQR)	3.3 (2.3–5.0)	2.7 (1.7–3.5)
Platelet-lymphocyte ratio (median, IQR)	149 (105–208)	156 (129–247)
Preoperative endoscopic biliary drainage, *n* (%)	121 (27)	17 (31)
Procedure (*n*, %)		
Pancreaticoduodenectomy (PD)	146 (33)	18 (33)
Pylorus preserving PD	107 (24)	21 (39)
Distal pancreatectomy	101 (23)	12 (22)
Total pancreatectomy	88 (20)	3 (6)
Tumor size, mm (median, IQR)	30 (25–40)	28 (22–44)
Perineural invasion, *n* (%)	292 (66)	34 (62)
Lymphovascular invasion, *n* (%)	339 (77)	39 (72)
Tumor differentiation, *n* (%)		
Well-differentiated	43 (10)	6 (11)
Moderately/poorly differentiated	399 (90)	48 (89)
AJCC stage (*n*, %)		
IA	18 (4)	3 (6)
IB	29 (7)	5 (9)
IIA	103 (23)	12 (22)
IIB	283 (64)	33 (61)
III	9 (2)	1 (2)
Resection margin, *n* (%)		
R0	226 (51)	29 (54)
R1/R2	210 (48)/6 (1)	24 (44)/1 (2)
Postoperative chemotherapy, *n* (%)	235 (53)	42 (78)

*IQR* interquartile range

### Blood Count Parameters

The median (IQR) values of blood count parameters are shown in Table 1. There were no significant differences in individual parameters between the retrospective and prospective patient cohorts. A correlation analysis failed to identify any association between blood count parameters and tumor characteristics, including tumor size, perineural invasion, lymphovascular invasion, grade, and TNM categories. There were no associations between blood counts and margin positivity. Moreover, blood count parameters did not influence rates of postoperative infective complications and mortality.

### Predictive Factors for Survival

**Fig. 1:**
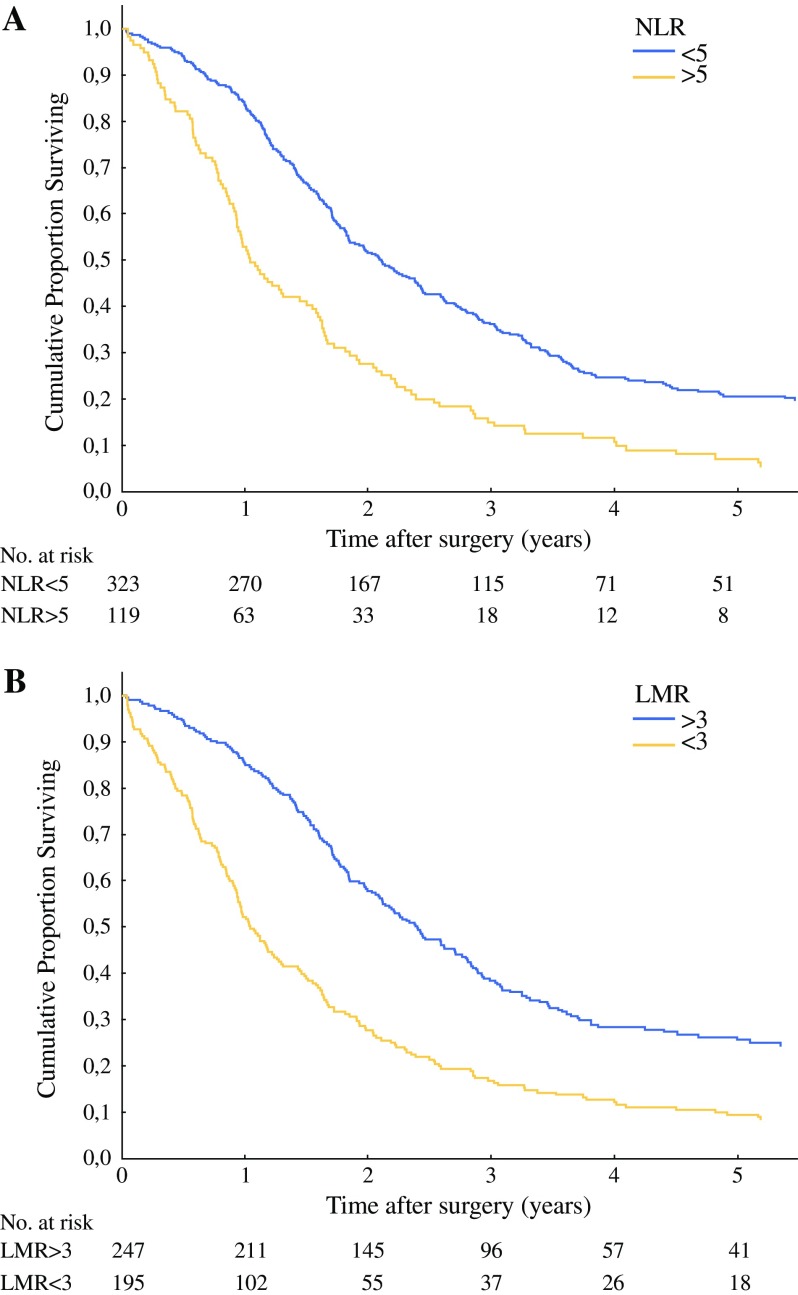
Kaplan–Meier curves of overall survival according to blood count parameters. **a** Neutrophil-lymphocyte ratio—NLR (log-rank test *P* = 0.001). **b** Lymphocyte-monocyte ratio—LMR (log-rank test *P* = 0.001)

Prognostic factors were analyzed in the *retrospective cohort*. Sixty-four of 442 patients were alive after a median follow-up of 93 months (range 15-290 months, final follow-up December 2015). The overall median survival was 19.2 months (95 % CI 14.1–24.2) with 3-year and 5-year survival rates of 28 and 16 %, respectively. Table 2 shows the results of the univariate analysis of potential prognostic factors. Among the three evaluated blood count parameters, the platelet-lymphocyte ratio did not influence survival either as a continuous or categorical variable. The neutrophil-lymphocyte ratio and lymphocyte-monocyte ratio were associated with survival and the time-dependent ROC curves analysis identified the best cut-off values to stratify patients into low and high risks of 5 and 3 for NLR and LMR, respectively. The overall median survival of patients with NLR ≥ 5 (*n* = 119) was significantly shorter than those with lower values of this ratio (12.6 vs 25.7 months, *P* = 0.001, Fig. 1a). Better median survival (29.2 vs 13.1 months, *P* = 0.001) was also found for high LMR values (LMR ≥ 3, *n* = 247, Fig. 1b). Subsequently, the Cox proportional hazards model showed that high NLR (HR 1.66, 95 % CI 1.12–2.46, *P* = 0.012) and low LMR (HR 1.65, 95 % CI 1.06–2.58, *P* = 0.026) were independent predictors of poor prognosis.

**Table 2:** Univariate and multivariate Cox proportional analysis for overall survival

Parameter	Categories^a^	Univariate analysis		Multivariate analysis	
		HR (95 % CI)	*P* ^b^	HR (95 % CI)	*P* ^c^
Age	<65/≥65 years	1.45 (1.07–1.96)	0.016	1.74 (1.19–2.55)	0.004
Gender	Female/male	1.07 (0.80–1.43)	0.662	–	
NLR	<5, ≥5	1.58 (1.12–2.72)	0.001	1.66 (1.12–2.46)	0.012
LMR	≥3, <3	1.93 (1.19–3.11)	0.001	1.65 (1.06–2.58)	0.026
CA 19-9 level	<180/> 180 U/mL	1.04 (0.68–1.41)	0.623	–	
Tumor location	Head/other	1.09 (0.77–1.54)	0.599	–	
Tumor size	<20/≥20 mm	1.08 (0.74–1.58)	0.675	–	
Perineural invasion	No/yes	1.41 (1.06–1.89)	0.018	1.32 (0.75–2.31)	0.332
Lymphovascular invasion	No/yes	0.98 (0.59–1.60)	0.935	–	
Tumor differentiation	Well/other	1.36 (0.81–2.28)	0.237	–	
AJCC T category	T1–2/T3–T4	2.25 (1.23–4.09)	0.008	2.90 (0.73–11.42)	0.127
AJCC N category	N0/N1	1.57 (1.13–2.17)	0.007	1.75 (1.090–2.79)	0.019
Metastatic lymph node ratio	<0.2; ≥0.2	1.92 (1.39–2.65)	<0.001	1.69 (1.10–2.60)	0.016
AJCC stage	I/II and III	1.86 (1.01–3.45)	0.049	2.96 (0.79–13.94)	0.337
Resection margin	R0/R1 and 2	2.17 (1.51–3.11)	<0.001	2.16 (1.16–4.02)	0.015
Chemotherapy	Yes/no	0.94 (0.61–1.45)	0.794	–	

*CI* confidence interval, *HR* hazard ratio, *NLR* neutrophil-lymphocyte ratio, *LMR* lymphocyte-monocyte ratio

^a^Reference category listed first

^b^Log Rank Mantel–Cox test

^c^Cox proportional hazards model

### Correlation of NLR and LMR with Changes in Lymphocyte and Monocyte Sub-populations

The hypothesis that NLR and LMR are associated with changes in lymphocyte and monocyte sub-populations was verified in the prospective cohort of patients. Twenty-two (41 %) patients showed low values of both NLR and LMR, 17 (31 %) had LMR ≥ 3, and elevated NLR was found in 15 (28 %) subjects. No patient demonstrated high values of both parameters. Table 3 shows the absolute counts of lymphocyte and monocyte populations according to the groups with high and low values of NLR and LMR. There were no significant differences in the proportions of T cells (78 %, IQR 73–81), helper T cells (47 %, IQR 40–53), cytotoxic T cells (24 %, IQR 20–33), B cells (9 %, IQR 7–10), and natural killer cells (12 %, IQR 8–17) in either group. However, the absolute counts differed significantly. NLR ≥ 5 was associated with nearly two-fold lower counts of all lymphocyte subpopulations. The group with low LMR (<3) showed similar alterations and additionally had elevated counts of all monocyte subsets compared to patients with LMR ≥ 3.

**Table 3:** Median (interquartile range) counts of lymphocyte and monocyte populations in groups with various NLR and LMR

Cell phenotype	Population	NLR		LMR	
		<5	≥5	<3	≥3
Neutrophils	Neutrophils	4250 (3257–6140)	6090 (4700–7570)*	4900 (3250–6200)	5300 (4000–7900)
Lymphocytes overall	Lymphocytes	1839 (1462–2550)	928 (484–1156)*	1316 (1026–1760)	2360 (1787–3948)*
CD3+	T cells	1384 (1007–1833)	761 (371–966)*	985 (788–1308)	1655 (1384–3055)*
CD3 +/CD4+	Helper T cells	797 (654–1252)	466 (271–581)*	589 (466–981)	808 (716–1811)*
CD3 +/CD8+	Cytotoxic T cells	421 (299–717)	244 (158–319)*	306 (223–373)	717 (485–1071)*
CD19+	B cells	164 (137–211)	66 (37–96)*	128 (66–180)	170 (137–211)*
CD16 + 56	NK cells	266 (148–314)	75 (40–143)*	174 (103–264)	314 (117–576)*
CD14 ++ CD16−	Monocytes, classical	669 (371–945)	512 (370–649)	659 (520–929)	395 (275–566)*
CD14 ++ CD16+	Monocytes, intermediate	26 (15–34)	22 (11–34)	29 (18–42)	23 (11–29)*
CD14 + CD16 ++	Monocytes, non-classical	43 (30–56)	35 (25–55)	49 (35–59)	34 (24–45)*

* *P* < 0.05 Mann–Whitney *U* test

## Discussion

Alterations in blood count parameters reflect the dynamic balance between anti-tumor and tumor-promoting functions of the immune system. The neutrophil-lymphocyte ratio and lymphocyte-monocyte ratio, simple derivatives of routine blood counts, were identified as important prognostic indicators in a large population dataset of patients with resected pancreatic cancer. Moreover, we have demonstrated that both these parameters are associated with changes in some populations of lymphocyte and monocyte subsets.

The idea of using inflammation-related prognostic scores utilizing blood count parameters has gained increasing attention in the treatment of various gastrointestinal malignancies, including pancreatic cancer.^14^–^17^ This was substantiated by the fact that such parameters were also associated with impaired survival in patients with advanced pancreatic cancer,^18^,^19^ including those receiving palliative chemotherapy^20^,^21^ and stereotactic body radiotherapy.^22^ Nevertheless, previous reports of resected pancreatic cancer were not uniform and were convoluted by some common problems, such as low number of patients, unclear selection of cut-off values,^23^–^25^ or concomitant evaluation of resectable and unresectable disease.^26^

Table 4 summarizes previous studies reporting prognostic applications of the three main parameters related to blood counts, i.e. NLR, LMR, and PLR. The majority of studies focused on NLR and the recruitment of large patient cohorts, including our findings, helped to show that high neutrophil-lymphocyte ratios (either ≥2, ≥3 or ≥5) significantly increased the risk of death. Although the precise mechanism is not completely understood, multifactorial effects of neutrophilia are postulated.^27^ An increased production of neutrophils is associated with higher proportions of immature cells and altered functional status. These changes are hypothesized to create an immunosuppressive milieu impairing the performance of the effector immune cells and promoting more aggressive growth of the tumor.^28^

**Table 4:** Summary of previous studies analyzing prognostic applications of NLR, LMR, and PLR in resected pancreatic cancer

Author, year	No. of patients	Cut-off values*	Median survival (months)	Multivariate survival analysis HR (95 % CI)
Neutrophil-lymphocyte ratio (NLR)				
Clark, 2007^23^	44	<5, ≥5	10.5/8.9	No effects on survival
Smith, 2009^4^	110	Continuous	Not applicable	No effects on survival
Bhatti, 2010^5^	84	Continuous	Not applicable	1.21 (1.01–1.45), *P* = 0.039
Jamieson, 2011^37^	135	<5, ≥5	20.9/25.7	No effects on survival
Garcea, 2011^38^	74	<5, ≥5	52.0/12.0	No effects on survival
Sanjay, 2012^39^	51	<5, ≥5	16.2/9.2	No effects on survival
Stotz, 2013^40^	110	<5, ≥5	Not reported	1.61 (1.02–2.53), *P* = 0.039
Hamed, 2013^41^	85	<5, ≥5	20.6/11.3	No effects on survival
Ben, 2015^42^	381	<2, ≥2	19.4/12.4	1.51 (1.15–1.99), *P* = 0.003
Watanabe, 2016^35^	46	<2.5, ≥2.5	Not reported	No effects on survival
Current study	442	<5, ≥5	25.7/12.6	1.66 (1.12–2.46), *P* = 0.012
Lymphocyte-monocyte ratio (LMR)				
Li, 2016^31^	144	<2.86, ≥2.86	12.0/19.0	0.15 (0.09–0.25), *P* < 0.001
Current study	442	≥3, <3	29.2/13.1	1.65 (1.06–2.58), *P* = 0.026
Platelet-lymphocyte ratio (PLR)				
Smith, 2009^4^	110	Continuous	Not applicable	1.004 (1.002–1.006), *P* < 0.001
Bhatti, 2010^5^	84	Continuous	Not applicable	No effects on survival
Jamieson, 2011^37^	135	<150, ≥150	26.7/20.7	No effects on survival
Sanjay, 2012^39^	51	150, 150–300, 300	15.9/15.0/4.1	No effects on survival
Stotz, 2013^40^	110	<150, ≥150	Not reported	No effects on survival
Watanabe, 2016^35^	46	<200, ≥200	Not reported	4.55, *P* = 0.002
Current study	442		No effects on survival	No effects on survival

* Reference category listed first

Monocytes are another important player involved in human malignancies. Although it is not yet certain which subset of the circulating monocyte pool, i.e. classical or non-classical, is recruited into the microenvironment as the main source of tumor-associated macrophages, these cells can exert local immunosuppressive effects promoting tumor growth and angiogenesis.^29^ Both high monocyte counts and low lymphocyte-to-monocyte ratios have been reported to be predictors of poor survival in patients with solid tumors.^30^ However, the importance of lymphocyte-to-monocyte ratio was evaluated only in a few studies recruiting patients with resectable pancreatic cancer. Li et al. in a group of 144 curatively-resected pancreatic cancers showed that LMR ≥ 2.86 was an independent favorable prognostic factor with HR 0.148 (95 % CI 0.085–0.252).^31^ Similar observations were reported in another study recruiting resectable and advanced cancers, where LMR > 2.8 reduced the risk of death (HR 0.81, 95 % CI 0.66–0.99, *P* = 0.040).^32^

An elevated platelet-lymphocyte ratio was associated with worse survival in various solid tumors.^33^ However, in contrast to NLR and LMR, high values of PLR impaired survival in only two reports involving small populations of resectable pancreatic cancer.^4^,^34^,^35^ As the prognostic implications of PLR were not also demonstrated in unresectable cases, it seems that this parameter is of limited significance.^20^

In a large and homogenous population of patients with resectable pancreatic cancer, we have demonstrated that high NLR and low LMR were associated with impaired long-term survival. However, unlike previous reports, we showed that these parameters were related to changes in populations of immune cells. Values of NLR and LMR implicated in worse prognosis correlated with an approximately twofold decrease in counts of all lymphocyte populations. High NLR was also accompanied by increased neutrophil counts, while low LMR showed increased numbers of monocytes, mostly classical. This clearly suggests that the unfavorable prognosis was likely correlated with reduced numbers of immune cells effective against the tumor and increased populations of cells involved in immune suppression.^27^,^36^

Our study has two potential limitations. First, lymphocyte and monocyte subpopulations were analyzed only in a small proportion of patients, even though there were no marked differences in most clinicopathologic parameters between the prospective and retrospective cohorts. Second, we did not evaluate the functional status of immune cells that could also contribute to impaired defense against cancer beside changes in the absolute counts. Therefore, future experiments should also consider the functional characteristics of the immune system in relation to altered blood counts.

In conclusion, this study demonstrated that variables obtained from routine blood count parameters may carry important prognostic information for patients with resected pancreatic cancer. High neutrophil-lymphocyte and low lymphocyte-monocyte ratios were associated with unfavorable prognosis. Moreover, both parameters were associated with marked alterations in the subsets of lymphocyte, monocyte, and neutrophil populations, likely contributing to impaired defense against cancer.
